# Homozygous mutation in *SLO3* leads to severe asthenoteratozoospermia due to acrosome hypoplasia and mitochondrial sheath malformations

**DOI:** 10.1186/s12958-021-00880-4

**Published:** 2022-01-03

**Authors:** Mingrong Lv, Chunyu Liu, Chunjie Ma, Hui Yu, Zhongmei Shao, Yang Gao, Yiyuan Liu, Huan Wu, Dongdong Tang, Qing Tan, Junqiang Zhang, Kuokuo Li, Chuan Xu, Hao Geng, Jingjing Zhang, Hang Li, Xiaohong Mao, Lei Ge, Feifei Fu, Kaixin Zhong, Yuping Xu, Fangbiao Tao, Ping Zhou, Zhaolian Wei, Xiaojin He, Feng Zhang, Yunxia Cao

**Affiliations:** 1 Reproductive Medicine Center, Department of Obstetrics and Gynecology, the First Affiliated Hospital of Anhui Medical University, Hefei, 230022 Anhui China; 2 NHC Key Laboratory of Study on Abnormal Gametes and Reproductive Tract (Anhui Medical University), Hefei, 230032 Anhui China; 3 Key Laboratory of Population Health Across Life Cycle, Anhui Medical University, Ministry of Education of the People’s Republic of China, Hefei, 230032 China; 4 Obstetrics and Gynecology Hospital, State Key Laboratory of Genetic Engineering at School of Life Sciences, Institute of Reproduction and Development, Fudan University, Shanghai, 200011 China; 5 Shanghai Key Laboratory of Female Reproductive Endocrine Related Diseases, Shanghai, 200011 China; 6 NHC Key Laboratory of Reproduction Regulation, Shanghai Institute for Biomedical and Pharmaceutical Technologies, Fudan University, Shanghai, 200011 China; 7 NHC Key Laboratory of Male Reproduction and Genetics, Guangdong Provincial Reproductive Science Institute (Guangdong Provincial Fertility Hospital), Guangzhou, 510600 China; 8 Department of Obstetrics and Gynecology, Fuyang Hospital of Anhui Medical University, Fuyang, China; 9 Anhui Province Key Laboratory of Reproductive Health and Genetics, Anhui Medical University, Hefei, China; 10 Anhui Provincial Human Sperm Bank, the First Affiliated Hospital of Anhui Medical University, Hefei, China

**Keywords:** Infertility, Asthenoteratozoospermia, *SLO3*, Acrosome hypoplasia, Mitochondrial sheath malformation

## Abstract

**Background:**

Potassium channels are important for the structure and function of the spermatozoa. As a potassium transporter, the mSlo3 is essential for male fertility as *Slo3* knockout male mice were infertile with the series of functional defects in sperm cells. However, no pathogenic variant has been detected in human *SLO3* to date. Here we reported a human case with homozygous *SLO3* mutation. The function of *SLO3* in human sperm and the corresponding assisted reproductive strategy are also investigated.

**Methods:**

We performed whole-exome sequencing analysis from a large cohort of 105 patients with asthenoteratozoospermia. The effects of the variant were investigated by quantitative RT-PCR, western blotting, and immunofluorescence assays using the patient spermatozoa. Sperm morphological and ultrastructural studies were conducted using haematoxylin and eosin staining, scanning and transmission electron microscopy.

**Results:**

We identified a homozygous missense variant (c.1237A > T: p.Ile413Phe) in the sperm-specific *SLO3* in one Chinese patient with male infertility. This *SLO3* variant was rare in human control populations and predicted to be deleterious by multiple bioinformatic tools. Sperm from the individual harbouring the homozygous *SLO3* variant exhibited severe morphological abnormalities, such as acrosome hypoplasia, disruption of the mitochondrial sheath, coiled tails, and motility defects. The levels of *SLO3* mRNA and protein in spermatozoa from the affected individual were reduced. Furthermore, the acrosome reaction, mitochondrial membrane potential, and membrane potential during capacitation were also afflicted. The levels of acrosome marker glycoproteins and PLCζ1 as well as the mitochondrial sheath protein HSP60 and SLO3 auxiliary subunit LRRC52, were significantly reduced in the spermatozoa from the affected individual. The affected man was sterile due to acrosome and mitochondrial dysfunction; however, intra-cytoplasmic sperm injection successfully rescued this infertile condition.

**Conclusions:**

*SLO3* deficiency seriously impact acrosome formation, mitochondrial sheath assembly, and the function of K^+^ channels. Our findings provided clinical implications for the genetic and reproductive counselling of affected families.

**Supplementary Information:**

The online version contains supplementary material available at 10.1186/s12958-021-00880-4.

## Introduction

Infertility is a significant global health issue affecting approximately 10–15% of couples worldwide, with the male factor being a component in at least 50% of couples. Studies have shown that many genetic and epigenetic factors play a role in male infertility, but many cases occur due to unknown reasons [1].

Many factors have been found to influence sperm motility and morphology, and genetic defects in the sperm result in the development of asthenoteratozoospermia [2]. Potassium channels are important factors in the structure and function of the sperm, and their defects can cause dysfunction in the physiology and function of sperm. As a potassium transporter, potassium channel subfamily U1 (*KCNU1*, also known as *SLO3*) has a high level of expression in the testis, and its deletion has been reported to lead to defects in sperm motility and fertility in mouse [3, 4]. In particular, SLO3, first cloned in mouse spermatocytes in 1998, belongs to the high conductance Slo K^+^ channels family and is a sperm-specific potassium channel whose physiological function has been confirmed by whole-cell patch clamping techniques [5]. Similar to SLO1, the calcium and voltage-gated potassium channel, SLO3 consists of a voltage-dependent pore formed by four identical α-subunits and a large cytoplasmic C-terminal, which has been proposed to be the ligand-binding sensor [6]. Both the pH and voltage dependence as well as the properties of the single channel of SLO3 have been well defined [7]. Despite the low sequence conservation of SLO3 among different mammalian species, it displays a high testis-specific expression pattern, located in the main part of the flagellum of sperm in mice [8, 9]. In addition, SLO3 has been reported to be sensitive to both pH and the voltage level and to play a role in sperm capacitation, sperm motility, and acrosome reaction. Deletion of the gene coding this ion channel might result in defective sperm activation and fertility in mice [3, 4, 10, 11].

So far, studies on the function of SLO3 have primarily focused on the mouse orthologue, mSLO3. Electrophysiological analysis and genetic deletion studies established that mSLO3 is a voltage- and pH-gated channel that mediates most K^+^ current in murine sperm. The function of mSLO3 has been shown to be essential for male fertility as *Slo3* knockout (KO) mice were found to be infertile, with sperm cells exhibiting a series of functional defects. In humans, it is unknown whether *SLO3* is functionally expressed in the sperm and serves a similar key role for the normal morphology and motility of the sperm and fertilization. Some reports have studied the properties of the hSLO3 channel by using the heterologous expression of hSLO3 channels in *Xenopus* oocytes [12, 13]. However, the exact functional properties of hSLO3 have not been characterized in human sperm. In this study, we analysed genetic data obtained by whole-exome sequencing (WES) from a large cohort of 105 patients with asthenoteratozoospermia, and identified a single patient carrying a homozygous missense mutation in *SLO3.* The adult male harbouring the homozygous *SLO3* variant was sterile, and characterized by abnormal sperm morphology and reduced sperm motility. However, intra-cytoplasmic sperm injection (ICSI) treatment using the mutant spermatozoa led to successful fertilization. Therefore, this work provided the first evidence that absence of *SLO3* causes male infertility due to abnormalities in sperm morphology, the mitochondrial sheath, acrosome reaction, and membrane potential of sperm in human.

## Materials and methods

### Subjects

In this study, we recruited a total of 105 Chinese men with asthenoteratozoospermia from the First Affiliated Hospital of Anhui Medical University. All 105 idiopathic infertile men were diagnosed with primary infertility for > 1 year. The individuals were recruited according to the guidelines of the World Health Organization (WHO) Laboratory Manual for the Examination and Processing of Human Semen [14]. Individuals with obvious primary ciliary dyskinesia-related symptoms, such as bronchitis, sinusitis, otitis media, or pneumonia, as well as infertility caused by reproductive malformation, drugs, or exposure to gonadotoxic factors, were excluded. Individuals with abnormalities in their karyotype (46,XY) or Y chromosome microdeletions were also excluded. Peripheral whole blood samples were collected from all recruited participants for subsequent genetic analysis.

### Ethical approval

This study was approved by the ethics committee of the First Affiliated Hospital of Anhui Medical University. Informed consent was obtained from all participants and their family members, as well as from all fertile control male individuals.

### Semen parameters and sperm morphological analysis

Semen samples from men with asthenoteratozoospermia and control subjects were collected via masturbation after 3–7 d of sexual abstinence and measured after liquefaction for 30 min at 37 °C in the source laboratories during the routine biological examination of individuals in accordance with the WHO guidelines (5th Edition) [14, 15]. Analyses of semen volume, sperm concentration, and motility were conducted during routine examination. Sperm morphology was analysed using haematoxylin and eosin (H&E) staining assay as previously described [16, 17], and 213 spermatozoa to evaluate the percentage of morphologically abnormal spermatozoa, such as small acrosome, swollen mid-piece, and coiled flagella. One spermatozoon was classified in only one morphological category according to its major abnormality.

### Structural modeling for SLO3 and its mutants

The mutant Ile413Phe of SLO3 is located near the Pfam motifs, the effect on protein structure were modelled with homology models. 3D structural model of the SLO3 mutant was carried out using UCSF Chimera software, based on the 4HPF.pdb protein template.

### Whole exome sequencing, bioinformatic analysis, and sanger sequencing

Genomic DNA was extracted from the peripheral blood of participants for WES analysis. Details on the methods used for library construction, WES, and data analysis were previously described [18]. The *SLO3* variant identified by WES was further validated by Sanger sequencing using the PCR primers presented in Table S1.

### Scanning and transmission electron microscopy

For the electron microscopy assay, semen samples were prepared in accordance with a protocol previously described [19]. Briefly, for scanning electron microscopy (SEM), samples were sequentially dehydrated using an ascending gradient of ethanol (Shengqiang Medical Technology, Jiangsu, China) and then dried with hexamethyldisilane (HMDS, Sigma-Aldrich, Castle Hill, NSW, Australia). Samples were then air-dried, added dropwise to specimen stubs, sputter coated, and examined using field emission SEM (Nova Nano 450, Thermo Fisher Scientific Inc., USA). For transmission electron microscopy (TEM), samples were fixed with 2.5% osmium tetroxide (Sigma-Aldrich, Castle Hill, NSW, Australia) and sequentially dehydrated using graded ethanol (50, 70, 90, and 100%) and 100% acetone (Sigma-Aldrich, Castle Hill, NSW, Australia). Samples were then infiltrated with acetone and SPI-Chem resin and embedded with Epon 812 (SPI#02659-AB, Structure Probe, USA). Subsequently, samples were sliced using an ultra-microtome (UC7, LEICA EM, Germany) and stained with uranyl acetate (#19481, Ted Pella, Inc., Redding, CA) and lead citrate (#19312, Ted Pella, Inc., Redding, CA). Cryoelectron microscopy (TecnaiG2 Spirit 120 kV, FEI, Netherlands) was used for image capturing.

### Quantitative real-time PCR and western blotting

Total RNA from human spermatozoa was isolated using the TRIzol Reagent (Invitrogen, Carlsbad, CA 92008 USA) and transcribed into cDNA using the PrimeScript RT Reagent Kit (Takara, Shiga, Japan) according to the manufacturer’s protocol. The obtained cDNAs were used as templates for subsequent quantitative real-time PCR conducted using the Light Cycler 480 SYBR Green I Master (Roche, Switzerland, Germany). *β-actin* was used as an internal control. The assays were repeated thrice. Primers used were listed in Table S2.

Proteins from human sperm samples for immunoblotting were extracted using the Mem-PER™ Plus Membrane Protein Extraction Kit (89,842, Thermo Fisher Scientific Inc., USA) according to the manufacturer’s instructions. Briefly, washed sperm samples were suspended in 0.5 mL permeabilization buffer, vortexed, and incubated for 10 min at 4 °C with constant mixing. Permeabilized sperm samples were then centrifuged for 15 min at 16000 *g* and the supernatant containing cytosolic proteins was transferred to a new tube. The pellet was resuspended in 0.5 mL solubilization buffer and incubated for 30 min at 4 °C with mixing. Finally, resuspended samples were centrifuged at 16000 *g* for 15 min at 4 °C, and the supernatant containing solubilized membranes was collected and heated at 100 °C for 15 min. Lysates were separated on 10% SDS-PAGE gels and transferred onto polyvinylidene fluoride (Pall Corporation, New York, NY, USA) membranes. Membranes were blocked in 5% non-fat milk diluted with TBST (TBS-0.1% Tween-20) for 1 h at 25 °C. Membranes were then immunoblotted using the following primary antibodies: rabbit polyclonal anti-LRRC52 (1:1000; PA5–107159, Invitrogen, Carlsbad, CA 92008 USA), rabbit polyclonal anti-CatSper1 (1:1000; DF9349, Affinity Biosciences, Beijing, China), rabbit polyclonal anti-Na^+^/K^+^-ATPaseα1 (1:2000; ABP51894, Abbkine, China), anti-HSP60 antibody (1:1000; ab13532, Abcam, Cambridge, UK), and HRP-conjugated β-actin (1:2000; HRP-60008, Proteintech, Rosemont, IL, USA), at 4 °C overnight. Signals were detected using the ECL Prime Western Blotting Detection Reagent (GE Healthcare, Beijing, China). Images were acquired using a CS analyser system (5200, Tanon, Shanghai, China). The Na^+^/K^+^-ATPase α1 or β-actin reference proteins were used as loading control.

### Generation of polyclonal anti-SLO3 antibody

SLO3 polyclonal antibodies were generated by ABclonal Biotechnology in New Zealand rabbits using the 1120–1134 and 1156–1170 polypeptides of the human SLO3 protein (ENSP00000382770) as antigens. Briefly, the cDNA encoding these epitopes was cloned into a pET-28a expression vector, and the His-tagged fusion protein was expressed in *Escherichia coli*. The purified recombinant protein was used to generate polyclonal antisera in female New Zealand rabbits. Sequences of the peptides used were as follows: 1. SYQPRTNSLSFPKQ 2. KENERKTSDEVYDED.

### Immunofluorescence assays

Immunofluorescence staining of sperm samples was performed as previously described [20]. Briefly, sperm samples were washed twice with phosphate buffer saline (PBS), fixed in 4% paraformaldehyde (Sigma-Aldrich, Castle Hill, NSW, Australia) at 4 °C overnight, and mounted on slides pre-treated with poly-L-lysine (Sigma-Aldrich, Castle Hill, NSW, Australia). Slides were incubated with primary antibodies (SLO3 (1:200, ABclonal Biotechnology, China), PLC-ζ1 (1:100, pab0367-P, covalab, USA), LRRC52 (1:500, PA5–107159, Invitrogen, Carlsbad, USA), CatSper1 (1:200; DF9349, Affinity Biosciences, Beijing, China), HSP60 (1:500; ab13532, Abcam, Cambridge, UK), acetylated alpha-tubulin (1:1000, mAb#5335, Cell Signaling Technology, Massachusetts, USA)) and PNA (1:500, RL-1072, VectorLabs, California, USA) overnight at 4 °C. After washing with PBS, slides were incubated with highly cross-adsorbed secondary Alexa Fluor 488 anti-mouse IgG (1:500, 34106ES60, Yeasen Biotechnology, USA) and Alexa Fluor 594 anti-rabbit IgG antibodies (1:500, 111–585-003, Jackson ImmunoResearch Inc., USA) for 1 h at 37 °C and subjected to Hoechst (1:1000, 62,249, Thermo Fisher Scientific Inc., USA) nuclear labelling for 2 h at 37 °C. Images were captured using an LSM 800 confocal microscope (CarlZeiss AG, Germany).

### Detection of mitochondrial membrane potential by flow cytometry acquisition

Measurements of mitochondrial membrane potential (MMP) were performed using the lipophilic cationic dye 5, 5′, 6, 6′-tetrachloro-1, 1′, 3, 3′-tetraethylbenzimidazolylcarbocyanineiodide (JC-1) according to the manufacturer’s instructions (Invitrogen). Briefly, approximately 1 million sperm cells were washed with PBS, followed by incubation with 5 μM JC-1 working solution for 15 min at 37 °C. Samples were then washed and analysed by flow cytometry. For further analysis of all cytometric experiments, the debris was gated out based on light scattering measurements. For each analysis, at least 50,000 sperm cells were re-examined. All experiments were performed on a BD FACSVerse™ Flow Cytometer (BD Biosciences, USA). Flow cytometry acquisition for JC-1-stained sperm cells was performed through FL1 for green and FL2 for red fluorescence. At high MMP, JC-1 forms J-aggregates inside the mitochondria emitting red fluorescence, whereas in a low MMP state, it remains in the monomer form emitting green fluorescence.

### Measurement of sperm membrane potential by flow cytometry

Sperm samples were washed twice with PBS. Pellets were then resuspended in Whitten’s HEPES-buffered media [(in mM): 135 NaCl, 5 KCl, 2 CaCl_2_, 1 MgSO_4_, 20 HEPES, 5 glucose, 10 lactic acid, 1 Na-pyruvate] with or without 25 mM NaHCO_3_ and 5 g/L BSA and incubated with 1 μM potential-sensitive dye 3, 3′-dipropylthiocabocyanine iodine (DiSC3, Sigma-Aldrich, Castle Hill, NSW, Australia) for 8 min at 37 °C. After incubation, PI (Sigma-Aldrich, Castle Hill, NSW, Australia) was added and incubated for further 3 min at 37 °C. Before assaying the sperm using flow cytometry, 500 nM carbonyl cyanide m-chlorophenylhydrazone (CCCP, Sigma-Aldrich, Castle Hill, NSW, Australia) was added in sperm suspensions and incubated at 37 °C for 2 min to dissipate the mitochondrial membrane potential. Analyses were conducted using a FACSVerse™ Flow Cytometer. Orange fluorescence from DiSC3-positive cells was detected at 600–700 nm and PI was detected at 500–560. Data were analyzed using FACS Diva and FlowJo software (Tree Star 9.3.3) as previously described [13]. Cell debris, doublets and aggregates were excluded from analysis based on a dual parameter dot plot, in which pulse signal (signal high; FSC-H; y-axis) versus signal area (FSC-A; x-axis) was displayed.

### ICSI procedures

The female partner of individual with *SLO3* mutation had undergone a long protocol pituitary downregulation using GnRH agonist (Triptorelin, Diphereline 3.75 mg, Ipsen Pharma Biotech) and control ovarian hyperstimulation by recombinant FSH (Gonal-F; Serono) Oestradiol plasma levels and follicle growth were monitored every 2 days and human chorionic gonadotrophin (HCG, Livzon Pharmaceutical) was administered when three or more than follicles reached 18 mm in diameter. 36 h after HCG injection, 15 mature oocytes (MII) were retrieved for ICSI. Spermatozoa were prepared by discontinuous density gradient centrifugation and the resulting suspension was diluted in 10 μl drops of polyvinyl pyrolidine (PVP) covered with oil. Subsequently, metaphase II stage oocytes and motile sperm were selected for ICSI using a micromanipulator system (Olympus, Japan). Eighteen to 19 h later, the fertilized oocytes were assessed and cultured in cleavage medium (Cook, USA) in an incubator with an environment of 37 °C, 5% O_2_, 6% CO_2_, and 89% N_2_ until day 3 after fertilization. Then, the evaluated embryos were transferred to blastocyst medium (Cook, USA) and incubated to day 5 or day 6. According to the scoring system of Gardner and Schoolcraft [21], we obtained three day-5 blastocysts (4AB, 4BB and 3BB) and nine day-6 blastocysts (4BA, 4BB, 4 BC, 3BB*2 and 3CC*3), and nine blastocysts (3CC*3 poor blastocysts were discarded) were cryopreserved to prevent ovarian hyperstimulation syndrome (OHSS). After 6 months, two day-5 blastocysts were thawed and transferred in successive artificial cycles and a single foetal heart beat was detected via ultrasound after 28 days.

### Statistical analyses

All data are representative of at least three independent experiments, and GraphPad Prism (GraphPad Software, San Diego, CA, USA) was used to perform the statistical analysis. Differences were analyzed by Student’s t-tests when comparing experimental groups, and *P*-values < 0.05 were considered significant.

## Results

### *SLO3* is involved in human male infertility with asthenoteratozoospermia

**Fig. 1:**
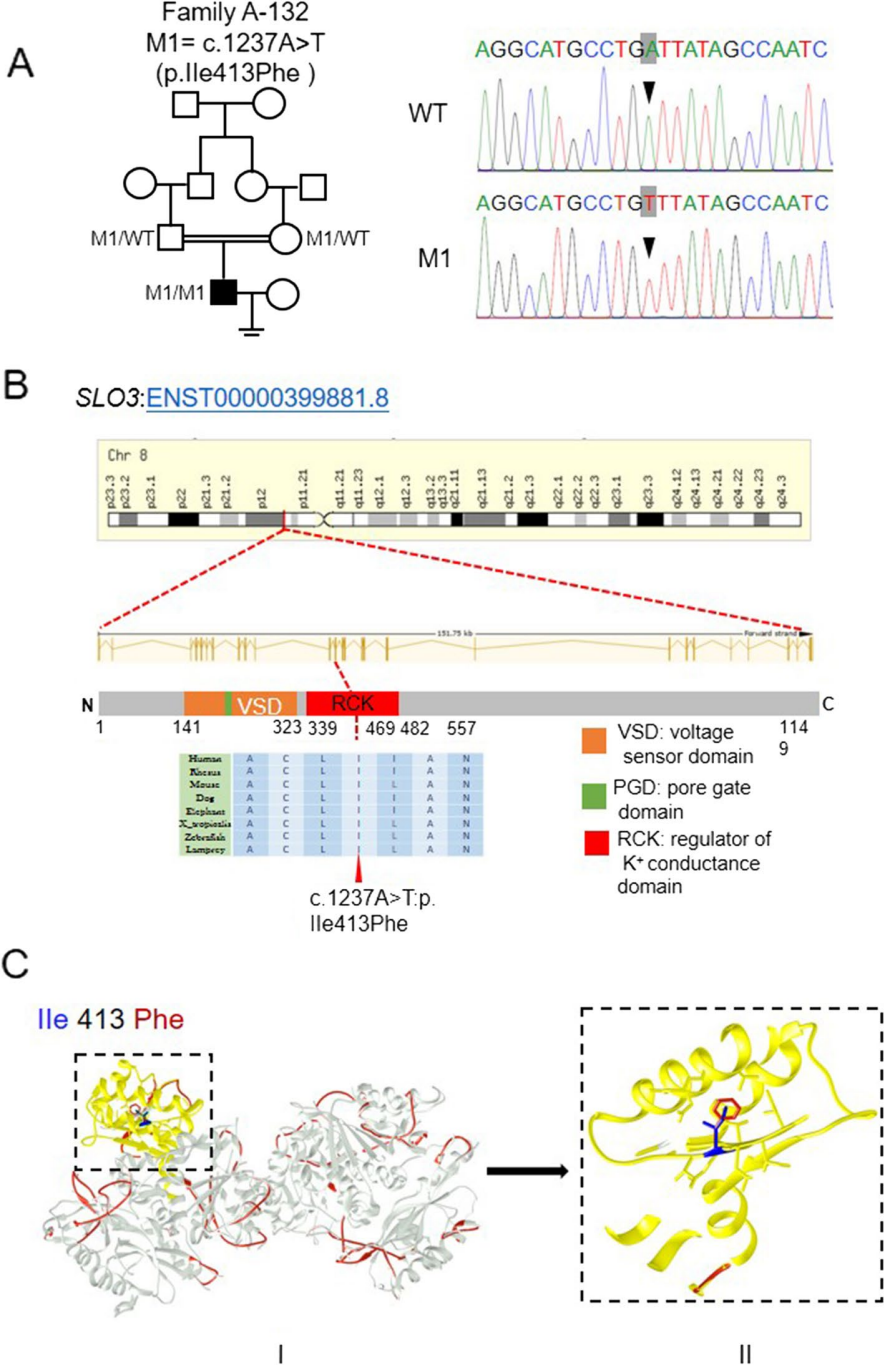
Identification of a homozygous mutation of *SLO3* in a Chinese man with asthenoteratozoospermia. **A** A homozygous missense mutation of *SLO3* (c.1237A > T), was identified in the proband from consanguineous family A-132. The amino acid alteration was predicted according to the verified alteration of cDNA. **B** The *SLO3* gene resides on chromosome 8, and the *SLO3* point mutation is located at the conserved site in the RCK domain. **C** Structure model of wild-type SLO3 protein (in silvery) and the mutant p. Ile (in blue) 413Phe (in red). The mutant residue (in red) is buried in the core of the RCK domain (in yellow). The mutant residue is bigger, probably not fitting and disturbing the core structure of this domain

We performed WES on a cohort of 105 individuals with severe asthenoteratozoospermia. After applying stringent filters criteria according to our previously described protocol, we identified a single individual (A-132) carrying a homozygous mutation in *SLO3* (GenBank: NM_001031836.3, c.1237A > T: p.Ile413Phe). The *SLO3* mutation was confirmed by Sanger sequencing (Fig. 1A). The subject A-132 from a consanguineous family, and presented with severely reduced sperm motility and a comparatively low sperm count (12.7 × 10^6^/mL) (Table 2, Fig. 1A). We noticed that the allele frequency of the *SLO3* variant was < 1% in any database, including 1000 Genomes Project and gnomAD (v2.1.1 with 141,456 samples). Moreover, the PolyPhen-2, SIFT, and MutationTaster tools predicted it to be deleterious (Table 1).

**Table 1:** Identification of Homozygous *SLO3* Variant in the Subject with Severe Asthenoteratozoospermia

***SLO3*** Variant Information	Subject A-132
cDNA alteration	c.1237A > T
Variant allele	Homozygous
Protein alteration	p. Ile413Phe
Variant type	Missense variant
**Allele Frequency in Human Populations**	
1KGP	0.0000122861
East Asians in gnomAD	0.0048076
All individuals in gnomAD	0.00004215
**Function Prediction**	
SIFT	damaging
PolyPhen-2	damaging
MutationTaster	disease causing

NCBI reference sequence accession number of *SLO3* is NM_001008723.2

*Abbreviations: 1KGP* 1000 Genomes Project, *gnomAD* Genome Aggregation Database

The *SLO3* (also known as *KCNU1*) gene is located on human chromosome 8 and contains 27 exons encoding a predicted 1149-amino acid potassium channel protein, specifically expressed in human spermatozoa. The SLO family channels are composed of 4 transmembrane voltage sensor domains (VSDs) surrounding a central pore gate domain (PGD), and a large cytosolic domain known as the gating ring. The gating ring is assembled from the regulators of K^+^ conductance (RCK1 and RCK2) domains of each of the 4 subunits, forming a ring with a central opening. The RCK domain determines the closed vs open conformational state of the gating ring and transduces the effects of any factor interacting with the gating ring to the PGD. Interestingly, the residue in the RCK domain of the SLO3 protein affected by the *SLO3* missense variant p. Ile413Phe is highly conserved in *SLO3* orthologues (Fig. 1B). Further analysis of protein structural modelling using online bioinformatic tools revealed the severe effects of this amino acid-substituting mutation on the structure of the SLO3 protein, indicating that this mutation introduces an amino acid with different properties, which may disturb this domain and abolish its function (Fig. 1C).

### Morphological abnormalities in sperm heads and mid-piece flagella of the man harbouring the homozygous *SLO3* variant

**Fig. 2:**
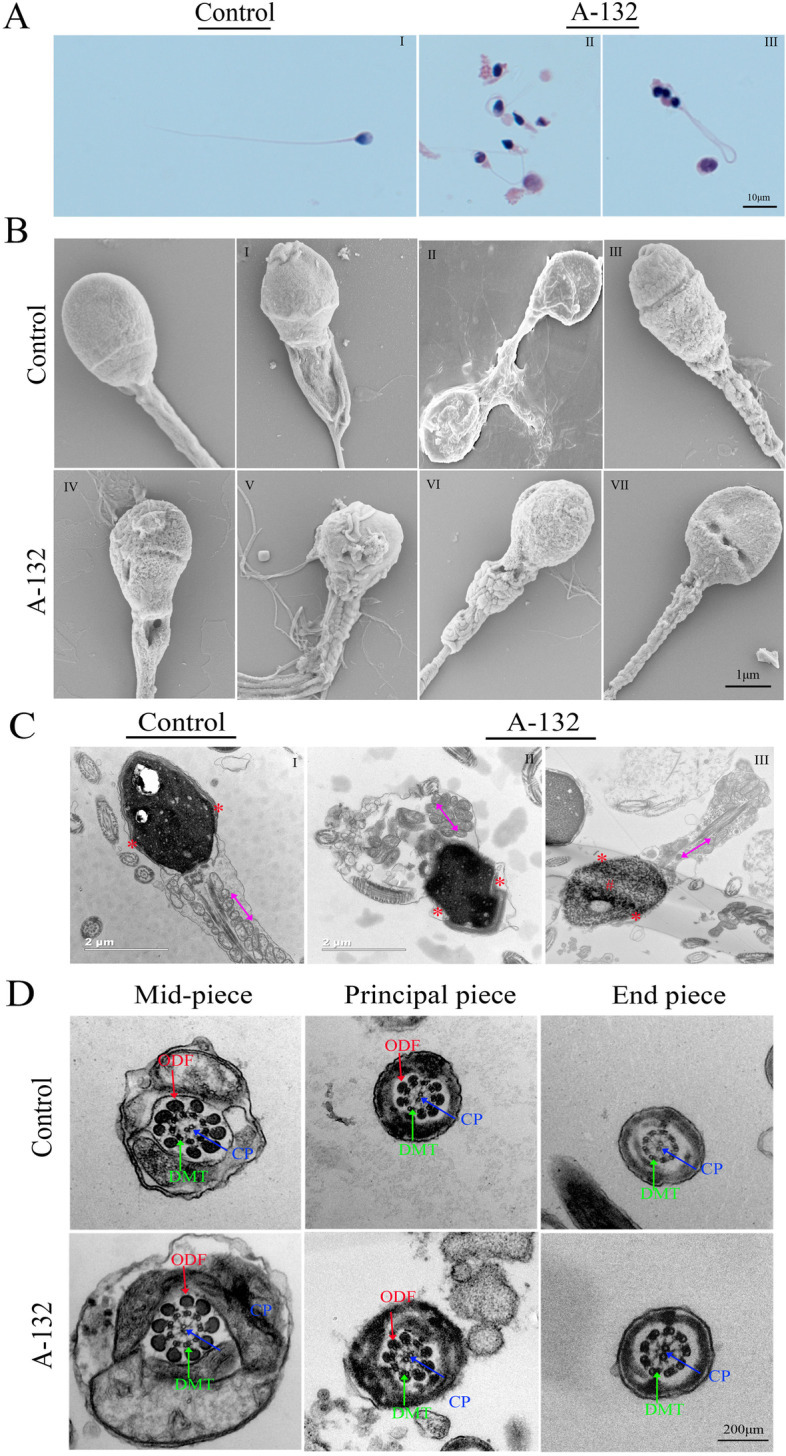
Sperm morphology and ultrastructure analyses in the spermatozoa from *SLO3*-mutated subject A-132. **A** Light microscopy analysis of spermatozoa from control (i) and the individual harbouring the *SLO3* variant. Most spermatozoa from the individual harbouring the *SLO3* variant have small acrosomal heads, swollen midpieces, and coil-shaped flagella. **B** SEM analyses of sperm cells from a fertile control and the *SLO3*-mutated individual (II-VIII). Spermatozoa of normal controls exhibit a smooth, regularly contoured, oval-shaped head and a flagellum with a clearly defined midpiece, whereas most sperm from the *SLO3* mutant individual exhibit severe morphological defects (II-VIII), such as swollen midpieces (II), coil-shaped flagella (III), small acrosome (IV), and absence of acrosome in combination with a defective midpiece (V-VIII). **C** TEM analyses of sperm cells from a fertile control and the *SLO3*-mutated individual. The longitudinal sections of spermatozoa from control and A-132. Sperm tails with poorly assembled mitochondria (II-red arrow) or a cytoplasmic mass containing different components of the flagellum (III-red arrow) are observed. The acrosome is thin or broken with unidentifiable acrosomal membranes (red asterisk) along with misshapen heads; chromatin condensation appears abnormal (number sign). **D** Cross-sections of the mid-piece, principal piece, and end piece of the flagella in a control individual show the typical “9 + 2” microtubule structure, including 9 peripheral microtubule doublets paired with 9 outer dense fibres and the central pair of microtubules, surrounded by the organized mitochondrial sheath or fibrous sheath. Ultrastructures of sperm from the *SLO3*-mutated individual are comparable to those from control. Scale bars: 10 μm (**A**), 1 μm (**B**), 2 μm (**C**), and 200 μm (**D**)

We examined sperm morphological and ultrastructural changes in sperm of *SLO3* mutant subject. Compared with the sperm from a fertile control individual, the spermatozoa from the individual harbouring the *SLO3* variant displayed a high rate of head malformations, in particular, an abnormal acrosomal region, indicating a defect in acrosome formation. In addition, we observed that sperm tails displayed swollen midpieces or shorter principal pieces of highly irregular width, indicating poorly assembled mitochondrial sheaths (Fig. 2A and Table 2). When observed by SEM, we found that spermatozoa of unaffected controls exhibited a smooth, regularly contoured, oval-shaped head and a long flagellum with a clearly defined midpiece, whereas most spermatozoa from the individual with the *SLO3* variant exhibited severe morphological defects (Fig. 2B), such as swollen midpieces (II and V), coil-shaped flagella (III), small acrosome (IV), absence of acrosome in combination with defective mid-piece (VI and VII), and insufficient chromatin condensation (VIII).

**Table 2:** Semen Parameters and Sperm Morphology of the *SLO3*-mutated Man

Subject	A-132	Reference Values
**Semen Parameters**		
Semen Volume (mL)	4.3	> 1.5^a^
Concentration (10^6^/mL)	**12.7**	> 15.0^a^
Motility (%)	**31.9**	> 40.0^a^
Progressive motility (%)	**4.8**	> 32.0^a^
**Sperm Morphology**		
Normal sperm (%)	**2.3** (5/215)	> 4.0^a^
Small acrosome (%)	**61.4** (132/215)	< 5.0^b^
Abnormal midpieces (%)	**35.3** (76/215)	< 11.4^c^
Coiled flagella (%)	**50.2** (108/215)	< 17.0^b^
Abnormal sperm (%)	**97.7**	<96^a^

^a^Reference limits according to the 5th WHO standards [14]

^b^Reference limits according to the distribution range of morphologically normal spermatozoa observed in 926 fertile subjects [17]

^c^Reference limits according to the distribution range of morphologically normal spermatozoa observed in 10 fertile subjects

At least 200 spermatozoa were observed for morphology analysis

To better understand the nature of these head and flagella defects observed in the spermatozoa with the *SLO3* mutation, we analysed their ultrastructure. We accordingly found that their acrosomes were either absent or morphologically defective (Fig. 2C). We also noticed that most acrosomes were quite thin, showing highly diminished contents with barely recognizable inner and outer acrosomal membranes. Furthermore, a higher incidence of insufficient chromatin condensation was also observed in the spermatozoa of the affected individual compared with those of controls. The longitudinal sections of the flagellar mid-piece of spermatozoa in the individual harbouring the homozygous *SLO3* variant showed a seriously disorganized or short mitochondrial sheath compared with those of control (Fig. 2C). However, we noticed that in cross-sections, the arrangements of ODF, DMT, and CP were not significantly different (Fig. 2D). Indeed, many mutant spermatozoa had a coil-shaped flagella that might have been associated with deficient osmoregulation and volume control, a condition expected to result from the mutation-induced ablation of the ion channel.

**Fig. 3:**
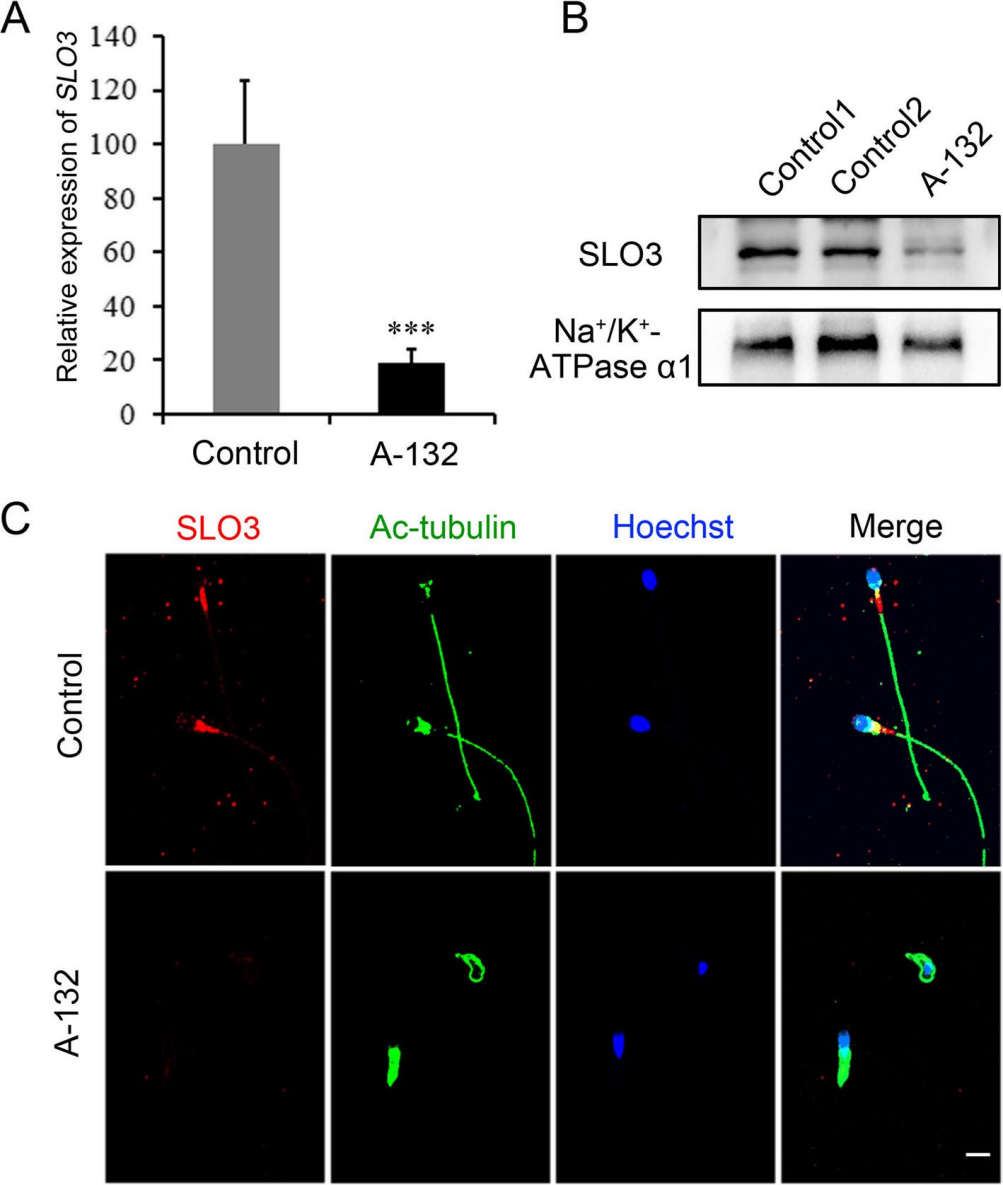
Expression analysis of *SLO3* mRNA and protein and localization of the SLO3 protein in sperm flagella. **A** Quantitative real-time PCR analysis of the expression of *SLO3* mRNA in the spermatozoa from normal control and the *SLO3*-mutated individual. Compared with control, the expression of *SLO3* mRNA is significantly reduced in the sperm from the *SLO3*-mutated individual. β-actin was used as an internal control. Data are presented as the mean ± SEM. ***P < 0.001; Student’s *t*-test. **B** Immunoblotting of sperm lysates from a normal control and the *SLO3*-mutated individual using an anti-SLO3 antibody. Na^+^/K^+^-ATPase α1 was used as loading control. **C** Representative images of spermatozoa from controls and from the *SLO3*-mutated individual stained with anti-SLO3 antibody, anti-Ac-tubulin antibody, and Hoechst. SLO3 staining is concentrated at the mid-piece of sperm flagella and faintly along the flagella in the fertile control but is significantly reduced in the sperm flagella of the individual harbouring the *SLO3* mutation. Scale bar, 20 μm

To further characterize the molecular defects induced by the *SLO3* mutation in human sperm, we examined the expression of *SLO3* mRNA and protein in sperm samples from control and individuals harbouring the *SLO3* variant. As shown in Fig. 3A, the *SLO3* mRNA was significantly (*P* < 0.001) reduced in spermatozoa from the man harbouring the *SLO3* variant, as well as the protein expression level (Fig. 3B and C), indicating that the observed sperm defects were probably induced by the deficiency in SLO3.

### *SLO3* deficiency affected the functions of acrosome and mitochondria

**Fig. 4:**
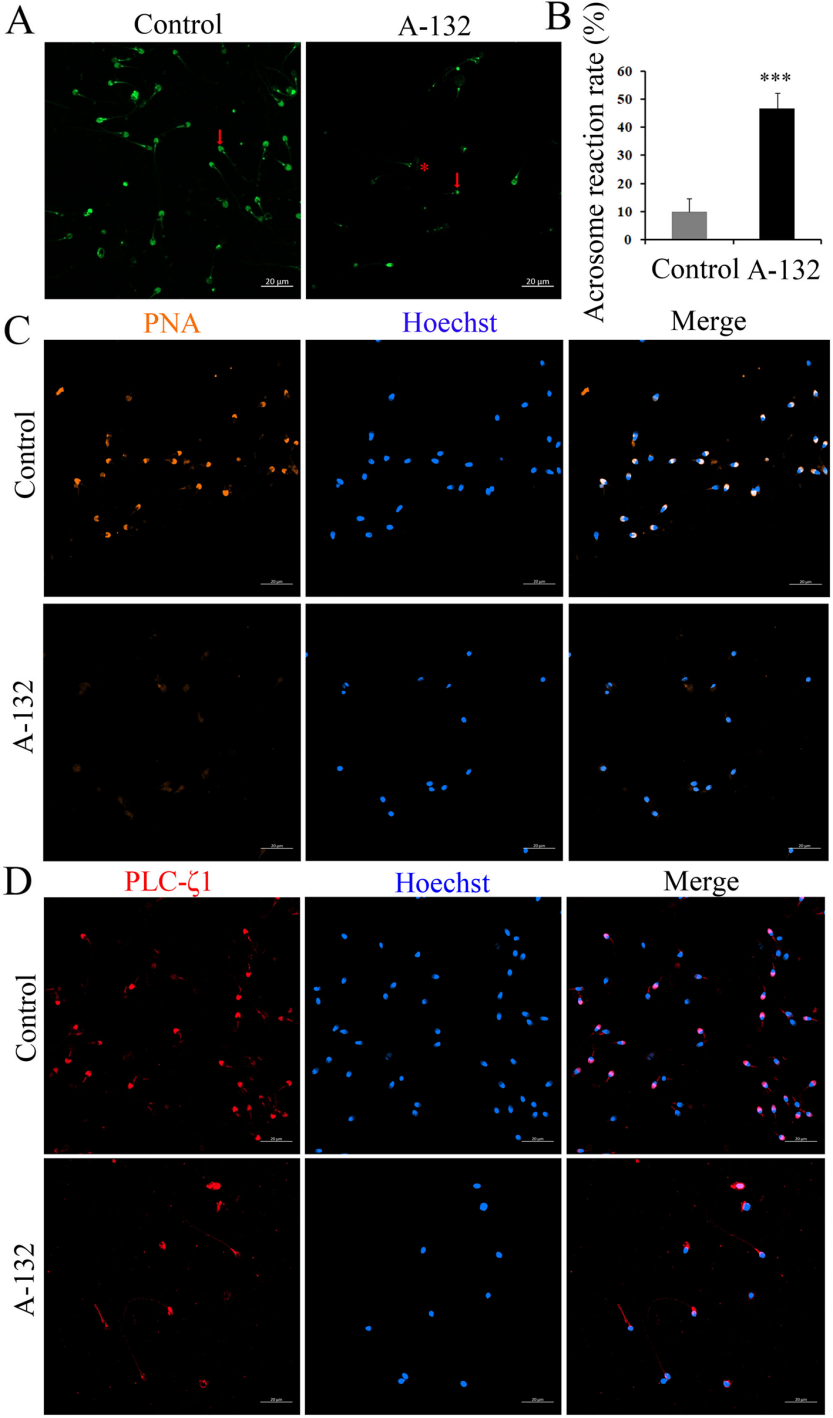
The acrosome reaction and expression patterns of acrosome are changed in the spermatozoa from *SLO3*-mutant man. **A** Influence of the *SLO3* mutation on spontaneous acrosome reaction. Staining with PSA-FITC: acrosome reacted spermatozoa (red asterisk); non-acrosome reacted spermatozoa (red arrowhead). **B** Percentage of spontaneous acrosome reaction. Data are presented as the mean ± SEM. Bars represent standard error. Significant differences, ***P < 0.001; Student’s *t*-test. **C** Peanut agglutinin (PNA) staining conjugated to rhodamine for the localization patterns of spermatozoa membrane galactose. **D** Localization patterns of PLCζ1 in normal and mutant sperm. Representative images of spermatozoa from controls and the *SLO3*-mutated individual stained with anti-PLCζ1 antibody, anti-Ac-tubulin antibody, and Hoechst. PLCζ1 staining is concentrated at the acrosomal region in control sperm, but absent in most mutant sperm, Scale bars: 20 μm

The ultrastructure analysis of spermatozoa from the individual harbouring the *SLO3* variant indicated that *SLO3* might play an essential role in acrosome formation and mitochondrial sheath assembly. To investigate the effect of the *SLO3* variant on the spermatozoa acrosome state, we evaluated the percentage of spontaneous acrosome-reacted cells using PSA-FITC binding. We found a significant increase (Student’s *t*-test; *P* < 0.001) in the rate of acrosome reaction in spermatozoa of the man harbouring the *SLO3* variant compared with that of controls (Fig. 4A and B). In addition, we also observed that most spermatozoa from controls presented a highly stained PNA in acrosomal region, whereas the signal was absent in most spermatozoa from the *SLO3* mutant individual. Moreover, the localization patterns of PLCζ1 were located in the acrosomal region in most spermatozoa from control individuals (Fig. 4C). However, spermatozoa from the subject harbouring the *SLO3* variant showed a predominant base of the neck and middle-piece of the tail localization pattern, indicating that both the formation of the acrosome and the localization pattern of PLCζ1 were influenced by the *SLO3* deficiency.

**Fig. 5:**
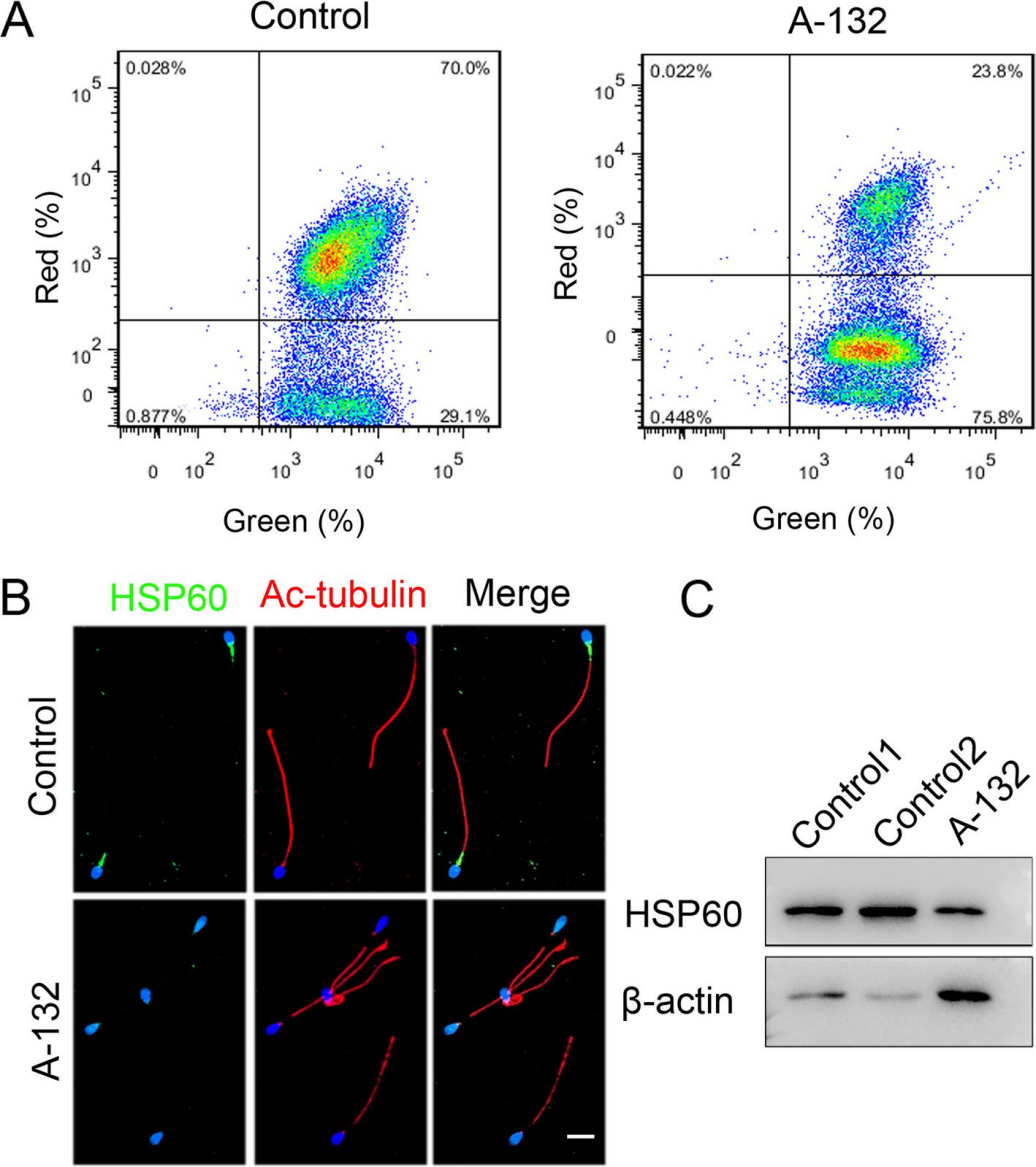
Effects of *SLO3* deficiency on mitochondrial function. **A** Influence of the *SLO3* mutation on mitochondrial membrane potential (MMP). Assessment of mitochondrial membrane potential by flow cytometry acquisition for JC-1-stained (a marker of MMP) cells was performed through FL1 for green and FL2 for red fluorescence. MMP is significantly reduced in mutant sperm compared with control. **B** Immunoblotting of sperm lysates from controls and the *SLO3*-mutated individual using the anti-HSP60 antibody. β-actin was used as loading control. **C** The expression of HSP60 is almost diminished in *SLO3* mutant spermatozoa. HSP60 staining is present in the flagellar midpiece of spermatozoa from normal controls, but significantly reduced in spermatozoa from A-132. Scale bars, 20 μm

Furthermore, we investigated the effect of *SLO3* deficiency on mitochondrial function. As shown in Fig. 5A, compared with control, the mitochondrial membrane potential was drastically decreased in spermatozoa from the man harbouring the *SLO3* variant. Subsequently, we examined the expression of HSP60 in the spermatozoa of the affected individual with that of healthy controls. We found that the expression of HSP60 was significantly reduced in the mitochondrial sheath components (Fig. 5B and C), in consistency with the sperm motility and the TEM results for spermatozoa of the affected individual. These results demonstrated that *SLO3* plays a key role in spermiogenesis by upholding the integrity of acrosomal and mitochondrial sheath components.

### The hyperpolarization of sperm from individual harbouring *SLO3* variant was decreased during capacitation

**Fig. 6:**
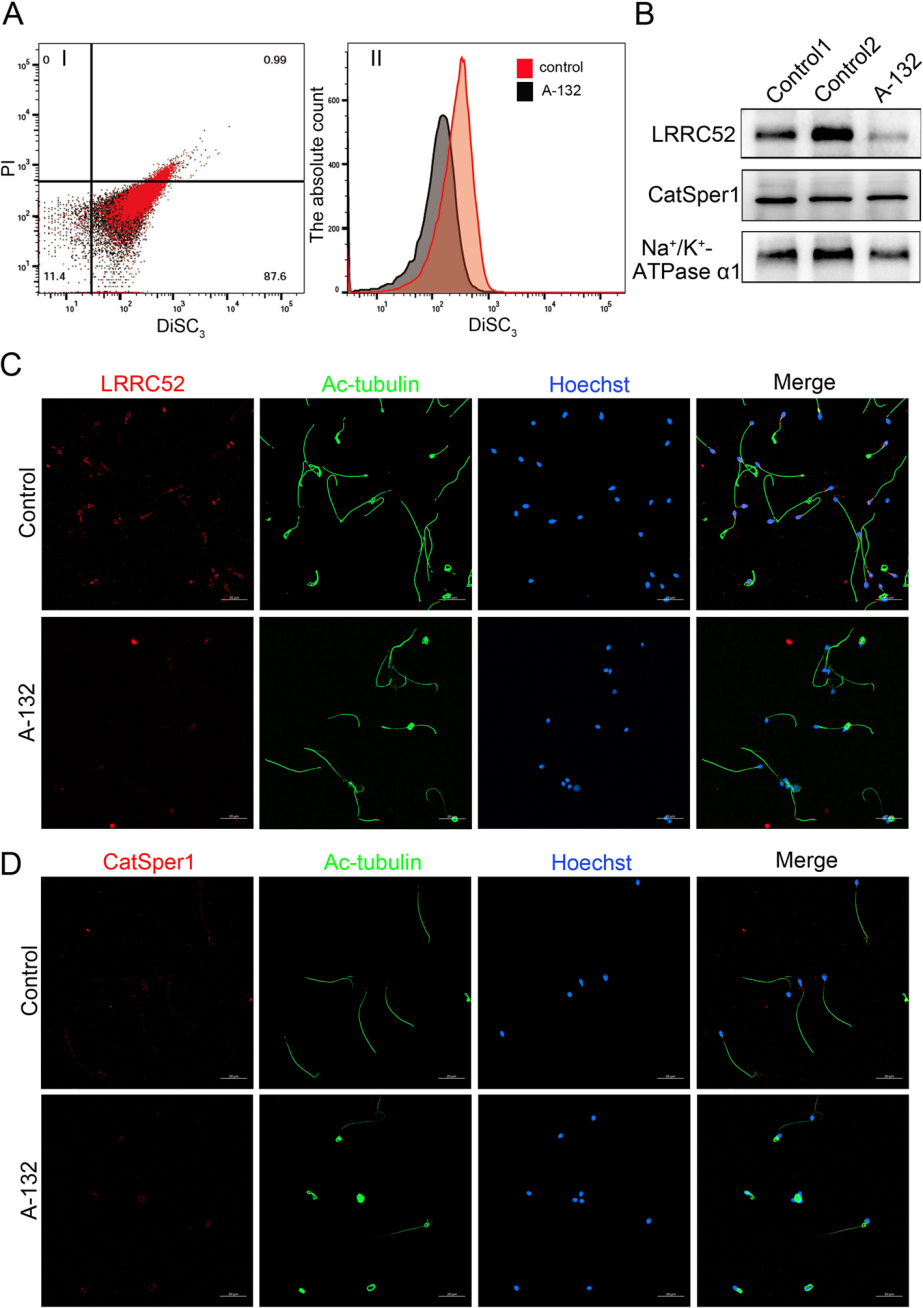
Membrane potential and expression patterns of LRRC52 and CatSper1 examined in the spermatozoa from control and *SLO3*-mutant subjects. **A** Membrane potential measurements of wild-type and *SLO3* mutant sperm in capacitated conditions. The capacitation-associated hyperpolarization of human sperm is inhibited in *SLO3* mutant spermatozoa, Data are presented as the mean ± SEM. ***P < 0.001; Student’s *t*-test. **B** Immunoblotting of sperm lysates from controls and the *SLO3*-mutated individual using the anti-LRRC52 and CatSper1 antibodies. Na^+^/K^+^-ATPase α1 was used as loading control. The expression of the LRRC52 protein is decreased in mutant sperm, whereas the level of the CatSper1 protein is comparable to that of control. **C** Representative images of spermatozoa from fertile controls and the *SLO3* mutant patient stained with the anti-LRRC52 antibody, anti-CatSper1 antibody, anti-Ac-tubulin antibody, and Hoechst. Two independent experiments were performed and at least 150 sperm we re-examined for each time per individual. Scale bars, 20 μm

In mouse sperm, deletion of the *Slo3* gene abolished alkalization-activated K^+^ current [4]. Thus, we wondered whether the *SLO3* homozygous mutant would alter the membrane potential of sperm during capacitation. Consistently, we found that the hyperpolarization of sperm from individual harbouring *SLO3* variant was decreased significantly during capacitation (Fig. 6A). To understand the molecular mechanism of the observed lack of membrane potential after capacitation in the *SLO3* mutant sperm, we examined the expression of the SLO3 auxiliary subunit LRRC52 in sperm cells from control and the *SLO3*-mutant individual Compared with normal controls (Fig. 6B and C), we noticed that the expression of LRRC52 was significantly decreased in sperm cells from the *SLO3*-mutant individual. Furthermore, we also investigated the sperm-specific change in CatSper expression in the spermatozoa of the *SLO3* mutated individual. We specifically observed that immunostaining of CatSper1 in the spermatozoa from the individual harbouring the *SLO3* variant was comparable to that observed in control sperm (Fig. 6B and D), suggesting that the Ca^2+^-activated K^+^ currents were controlled by LRRC52 and not CatSper1.

### SLO3 deficiency-related male infertility could be rescued by intracytoplasmic sperm injection treatment

It has been previously reported that *Slo3*^*−/−*^ male mice are infertile, but *Slo3*^*−/−*^ sperm exhibits some fertility when utilized in vitro fertilization assays. In addition, ICSI has been suggested as an effective way to circumvent the physical limitations experienced by asthenoteratozoospermia sperm in our hospital. The individual with the *SLO3* variant was 29 years old at treatment, while his partner was 34 years old. As a couple, they underwent 1 ovarian stimulation cycle, with the number of metaphase II oocytes retrieved and injected being 15. We observed that the rates of the fertilization, cleavage, 8-cell and blastocyst formation ware comparable to those in OAT controls (Table 3). Consecutively, 9 blastocysts were frozen. Six months later, 2 blastocysts were transferred and a single foetal heart beat was detected via ultrasound, with the pregnancy being currently ongoing. Therefore, our study indicated that male infertility caused by a homozygous *SLO3* variant could be rescued by ICSI treatment.

**Table 3:** Clinical outcomes of ICSI for the *SLO3*-mutated Man

	***SLO3***-mutated Man	OAT as Controls
Male age (years)	29	30.1 ± 4.9
Female age (years)	34	28.4 ± 4.2
No. of ICSI cycles	1	301
No. of oocytes injected	15	11 (1, 34)
Fertilization rate (%) (and number)	100 (15/15)	83.3 (12.5, 100.0) (3219/4077)
Cleavage rate (%) (and number)	100 (15/15)	100.0 (66.7, 100.0) (3188/3219)
8-Cell formation rate (%) (and number)	73 (11/15)	70.6 (10.5, 100.0)
Blastocyst formation rate (%) (and number)	73 (11/15)	60.0 (0, 100.0)
No. of transfer cycles	1	301
Number of embryos transferred per cycle	2	2 (1, 2)
Implantation rate (%)	50	47.9 (229/478)
Clinical pregnancy rate (%)	100	57.1 (172/301)
Miscarriage rate (%)	0	11.6 (20/172)

*OAT* Oligoasthenoteratozoospermia; The number of OAT couples is 301

## Discussion

To the best of our knowledge, we demonstrated for the first time that the presence of a homozygous mutation of *SLO3* could induce severe morphological defects in the head and midpiece of human spermatozoa, together with a deficiency in the acrosome reaction and impaired progressive motility in sperm. The SLO3 protein localizes in the flagellum and is especially concentrated at the flagellar midpiece of spermatozoa, possibly playing an important role in mitochondrial sheath assembly. In addition, when observed by light microscopy and SEM, most spermatozoa from the individual harbouring the *SLO3* variant exhibited angular/bent tails compared with those of healthy controls, consistent with the observations in *Slo3*^*−/−*^ mouse sperm [4]. However, in cross-sections, the arrangements of ODF, DMT, and CP were not significantly different, which might have been associated with a deficient osmoregulation and volume control in sperm, a condition probably resulting from the mutation-induced ablation of a major ion channel [4].

The eukaryotic SLO1 and SLO3 proteins exhibit a high amino acid sequence identity (42% identity), and, in fact, sequence analysis has suggested that *SLO3* evolved from a duplication of the *SLO1* gene [6, 22]. However, SLO1 and SLO3 channels share a unique property among the extended family of voltage-gated K^+^ channels in that their opening requires the synergistic action of membrane depolarization and specific intracellular cues [6]. The *SLO1* gene is expressed in excitable cells, such as neurons or muscles [5]. Whereas *SLO3*, which is exclusively expressed in mammalian sperm, is a voltage- and pH-gated channel that mediates most K^+^ current in murine sperm essential to male fertility and is activated by an increase in intracellular pH [3, 23]. In humans, the exact functional properties of hSLO3 have not been characterized. By heterologously expressing human *SLO3* in *Xenopus* oocytes, MacKinnon et al. found that the pH sensitivities of hSLO3 and mSLO3 were comparable [6]. Whereas Strünker et al. observed that the membrane potential in human sperm was weakly activated by pH and more strongly by Ca^2+^, a finding that differed from that observed for the mSlo3 channel [12]. In our study, the *SLO3* mutation impaired the membrane potential of mutant sperm after capacitation. In addition, the expression of the SLO3 auxiliary subunit LRRC52 was significantly reduced in sperm from the *SLO3* mutant individual compared with that from control, in accordance with the finding that LRRC52 was completely absent from *Slo3*^*−/−*^ sperm [24]. This result indicated that the expression of LRRC52 is critically dependent on the presence of SLO3 [24–26]. Moreover, the expression of CatSper1 was similar in both control and effected sperm, indicating that Ca^2+^ channels were not affected by the mutation in *SLO3*.

It has been reported that *Slo3*^*−/−*^ male mice were unable to fertilize wild-type females, despite the presence of fertilization plugs [4]. However, partial IVF success was achieved with oocytes harvested from super ovulating wild-type females incubated with capacitated sperm from *Slo3*^*−/−*^ male mice [3]. Besides, ICSI has become an effective method to help infertile couples, particular for infertile men with asthenoteratozoospermia, achieve a successful pregnancy. In this study, we noticed that the fertilization, cleavage, 8-cell and blastocyst formation rates were similar when performing ICSI with sperm from individual harbouring *SLO3* variant and control sperm. We froze 9 blastocysts and 6 months later, transferred 2 blastocysts, resulting in the detection of a single foetal heartbeat via ultrasound; the pregnancy is ongoing. Therefore, our study indicated that male infertility caused by a homozygous *SLO3* variant could be rescued by ICSI treatment, which is meaningful for the genetic counselling of patients with *SLO3*-associated asthenoteratozoospermia.

## Conclusion

In conclusion, our findings on the homozygous mutation of *SLO3* might further broaden our knowledge on genetic pathologies associated with male infertility due to severe asthenoteratozoospermia. Our findings might also have important clinical implications for the genetic and reproductive counselling of affected families. Deletion of *SLO3* would seriously impact acrosome formation, mitochondrial sheath assembly, and the function of K^+^ channels. However, there were certain limitations in the present study. Further investigations identifying the functional role of *SLO3* in acrosome formation, mitochondrial sheath assembly are required, and such investigations might involve the screening of a large number of patients with severe asthenoteratozoospermia.

## Supplementary Information

**Additional file 1: Table S1.** Primers Used for Amplification and Verification of *SLO3* Mutations. **Table S2.** Primers Used for qRT-PCR Assays Primer.

## Data Availability

The datasets used and/or analysed during the current study are available from the corresponding author on reasonable request.

## Abbreviations

WES: Whole-exome sequencing
HE: Haematoxylin and eosin
SEM: Scanning electron microscopy
TEM: Transmission electron microscopy
ICSI: Intra-cytoplasmic sperm injection
qRT-PCR: Quantitative real-time PCR

## References

[1] Miller D, Vukina J (2020). Recent advances in clinical diagnosis and treatment of male factor infertility. Postgrad Med.

[2] Saberiyan M, Mirfakhraie R, Gholami D, Dehdehi L, Teimori H (2020). Investigating the regulatory function of the ANO1-AS2 on the ANO1 gene in infertile men with asthenozoospermia and terato-asthenozoospermia. Exp Mol Pathol.

[3] Santi CM, Martinez-Lopez P, de la Vega-Beltran JL, Butler A, Alisio A, Darszon A, Salkoff L (2010). The SLO3 sperm-specific potassium channel plays a vital role in male fertility. FEBS Lett.

[4] Zeng XH, Yang C, Kim ST, Lingle CJ, Xia XM (2011). Deletion of the Slo3 gene abolishes alkalization-activated K+ current in mouse spermatozoa. Proc Natl Acad Sci U S A.

[5] Schreiber M, Wei A, Yuan A, Gaut J, Saito M, Salkoff L (1998). Slo3, a novel pH-sensitive K+ channel from mammalian spermatocytes. J Biol Chem.

[6] Leonetti MD, Yuan P, Hsiung Y, Mackinnon R (2012). Functional and structural analysis of the human SLO3 pH- and voltage-gated K+ channel. Proc Natl Acad Sci U S A.

[7] Zeng XH, Navarro B, Xia XM, Clapham DE, Lingle CJ (2013). Simultaneous knockout of Slo3 and CatSper1 abolishes all alkalization- and voltage-activated current in mouse spermatozoa. J Gen Physiol.

[8] Geng Y, Ferreira JJ, Dzikunu V, Butler A, Lybaert P, Yuan P, Magleby KL, Salkoff L, Santi CM (2017). A genetic variant of the sperm-specific SLO3 K(+) channel has altered pH and ca (2+) sensitivities. J Biol Chem.

[9] Santi CM, Butler A, Kuhn J, Wei A, Salkoff L (2009). Bovine and mouse SLO3 K+ channels: evolutionary divergence points to an RCK1 region of critical function. J Biol Chem.

[10] De La Vega-Beltran JL, Sanchez-Cardenas C, Krapf D, Hernandez-Gonzalez EO, Wertheimer E, Trevino CL, Visconti PE, Darszon A (2012). Mouse sperm membrane potential hyperpolarization is necessary and sufficient to prepare sperm for the acrosome reaction. J Biol Chem.

[11] Demarco IA, Espinosa F, Edwards J, Sosnik J, De La Vega-Beltran JL, Hockensmith JW, Kopf GS, Darszon A, Visconti PE (2003). Involvement of a Na+/HCO-3 cotransporter in mouse sperm capacitation. J Biol Chem.

[12] Brenker C, Zhou Y, Muller A, Echeverry FA, Trotschel C, Poetsch A, Xia XM, Bonigk W, Lingle CJ, Kaupp UB, Strunker T (2014). The Ca2+−activated K+ current of human sperm is mediated by Slo3. Elife.

[13] Lopez-Gonzalez I, Torres-Rodriguez P, Sanchez-Carranza O, Solis-Lopez A, Santi CM, Darszon A, Trevino CL (2014). Membrane hyperpolarization during human sperm capacitation. Mol Hum Reprod.

[14] Cooper TG, Noonan E, von Eckardstein S, Auger J, Baker HW, Behre HM, Haugen TB, Kruger T, Wang C, Mbizvo MT, Vogelsong KM (2010). World Health Organization reference values for human semen characteristics. Hum Reprod Update.

[15] Bjorndahl L, Barratt CL, Mortimer D, Jouannet P (2016). ‘How to count sperm properly’: checklist for acceptability of studies based on human semen analysis. Hum Reprod.

[16] He X, Liu C, Yang X, Lv M, Ni X, Li Q, Cheng H, Liu W, Tian S, Wu H (2020). Bi-allelic loss-of-function variants in CFAP58 cause flagellar Axoneme and mitochondrial sheath defects and Asthenoteratozoospermia in humans and mice. Am J Hum Genet.

[17] Auger J, Jouannet P, Eustache F (2016). Another look at human sperm morphology. Hum Reprod.

[18] Tang S, Wang X, Li W, Yang X, Li Z, Liu W, Li C, Zhu Z, Wang L, Wang J (2017). Biallelic mutations in CFAP43 and CFAP44 cause male infertility with multiple morphological abnormalities of the sperm flagella. Am J Hum Genet.

[19] Liu C, He X, Liu W, Yang S, Wang L, Li W, Wu H, Tang S, Ni X, Wang J (2019). Bi-allelic mutations in TTC29 cause male subfertility with Asthenoteratospermia in humans and mice. Am J Hum Genet.

[20] He X, Li W, Wu H, Lv M, Liu W, Liu C, Zhu F, Li C, Fang Y, Yang C (2019). Novel homozygous CFAP69 mutations in humans and mice cause severe asthenoteratospermia with multiple morphological abnormalities of the sperm flagella. J Med Genet.

[21] Gardner DK, Schoolcraft WB (1999). Culture and transfer of human blastocysts. Curr Opin Obstet Gynecol.

[22] Vicens A, Vinuesa P, Arenas M, Trevino CL (2019). Analyzing the functional divergence of Slo1 and Slo3 channel subfamilies. Mol Phylogenet Evol.

[23] Zhang X, Zeng X, Lingle CJ (2006). Slo3 K+ channels: voltage and pH dependence of macroscopic currents. J Gen Physiol.

[24] Yang C, Zeng XH, Zhou Y, Xia XM, Lingle CJ (2011). LRRC52 (leucine-rich-repeat-containing protein 52), a testis-specific auxiliary subunit of the alkalization-activated Slo3 channel. Proc Natl Acad Sci U S A.

[25] Kawai T, Miyata H, Nakanishi H, Sakata S, Morioka S, Sasaki J, Watanabe M, Sakimura K, Fujimoto T, Sasaki T (2019). Polarized PtdIns (4,5) P2 distribution mediated by a voltage-sensing phosphatase (VSP) regulates sperm motility. Proc Natl Acad Sci U S A.

[26] Zeng XH, Yang C, Xia XM, Liu M, Lingle CJ (2015). SLO3 auxiliary subunit LRRC52 controls gating of sperm KSPER currents and is critical for normal fertility. Proc Natl Acad Sci U S A.

