# Supplementary material for: Homozygous mutation in SLO3 leads to severe asthenoteratozoospermia due to acrosome hypoplasia and mitochondrial sheath malformations
Source: Reprod Biol Endocrinol. 2022 Jan 3;20:5. doi: 10.1186/s12958-021-00880-4 (PMC8722334; doi:10.1186/s12958-021-00880-4)
Supplement: Supplementary file 1 — Additional file 1: Table S1. Primers Used for Amplification and Verification of SLO3 Mutations. Table S2. Primers Used for qRT-PCR Assays Primer. [file 12958_2021_880_MOESM1_ESM.docx]

**Table S1. Primers Used for Amplification and Verification of *SLO3* Mutations**

| **Primer Names** | **Primer Sequences (5'-3')** | **Tm** |
| --- | --- | --- |
| M1-F | CCAAGTAGACTCCCAGTTCC | 57℃ |
| M1-R | ACACTCAGGATGCTCCGTTT |  |

**Table S2. Primers Used for qRT-PCR Assays Primer**

| **Primer Names** | **Primer Sequences (5'-3')** | **Tm** |
| --- | --- | --- |
| H-*SLO3*-F | ACGTTCATTTCTGGATCTGC | 60℃ |
| H-*SLO3*-R | TGATAGAGAGCACCCTCATA |  |
